# *Lutzomyia adiketis *sp. n. (Diptera: Phlebotomidae), a vector of *Paleoleishmania neotropicum *sp. n. (Kinetoplastida: Trypanosomatidae) in Dominican amber

**DOI:** 10.1186/1756-3305-1-22

**Published:** 2008-07-15

**Authors:** George Poinar

**Affiliations:** 1 Department of Zoology, Oregon State University, Corvallis, Oregon, 97331, USA

## Abstract

**Background:**

Amber fossils can be used to trace the history of disease-vector associations because microorganisms are preserved "in situ" inside the alimentary tract and body cavity of blood-sucking insects.

**Results:**

*Lutzomyia adiketis *sp. n. (Phlebotomidae: Diptera) is described from Dominican amber as a vector of *Paleoleishmania neotropicum *sp. n. (Kinetoplastida: Trypanosomatidae). The fossil sand fly differs from all previously described extinct and extant members of the genus by the following combination of characters: Sc forked with the branches meeting the costa and radius veins; wing L/W value of 4.1; a δ value of 18; a ratio β/α value of 0.86, and the shape and size of the spatulate rods on the ninth sternite. The trypanosomatid is characterized by the structure of its promastigotes, amastigotes and paramastigotes and its transmission by an extinct species of sand fly.

**Conclusion:**

Morphological characters show that the fossil sand fly is a new extinct species and that it is host to a digenetic species of trypanosomatid. This study provides the first fossil evidence that Neotropical sand flies were vectors of trypanosomatids in the mid-Tertiary (20–30 mya).

## Background

Moth flies (Psychodidae) and sand flies (Phlebotomidae) are primitive Diptera [1] often treated as subfamilies [2]. The fossil record of sand flies dates back to Early Cretaceous Lebanese [3,4] and Burmese amber [5]. The Burmese amber sand fly, *Palaeomyia burmitis *Poinar [5] was transmitting *Paleoleishmania protera *Poinar & Poinar [6,7], the first described fossil digenetic trypanosomatid parasite. The present study describes a second species of *Paleoleishmania *carried by an extinct species of *Lutzomyia *sand fly in Dominican amber.

## Results

### Description of vector

Family Phlebotomidae Kertész 1903

Genus *Lutzomyia *França 1924

**Figure 1:**
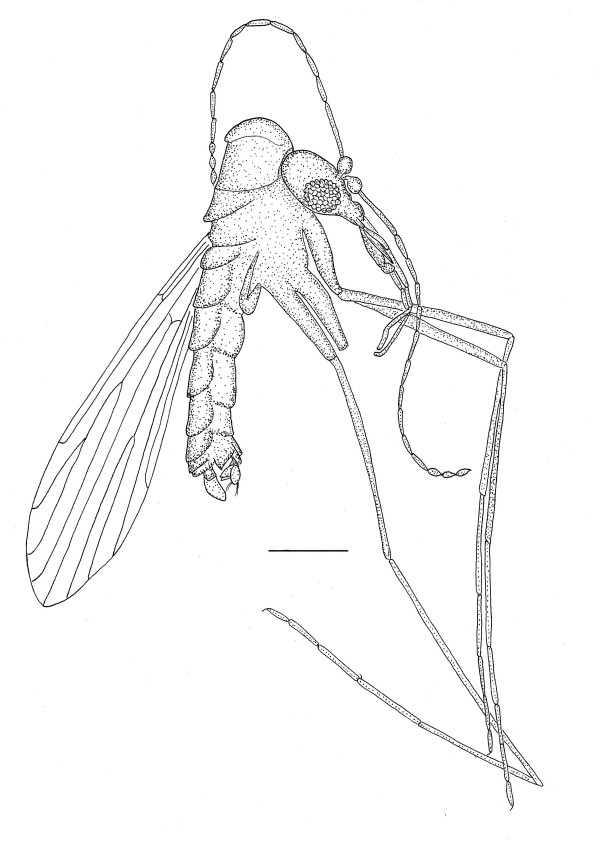
**Drawing of *Lutzomyia adiketis *showing wing venation.** Bar = 270 μm.

**Figure 2:**
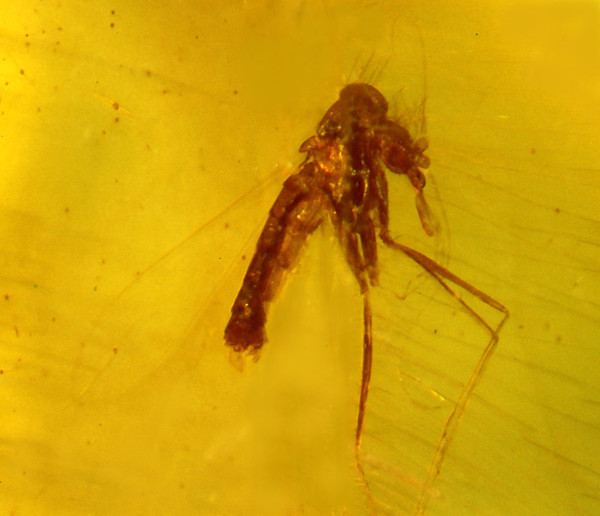
**Habitus of *Lutzomyia adiketis*.** Bar = 240 μm.

**Figure 3:**
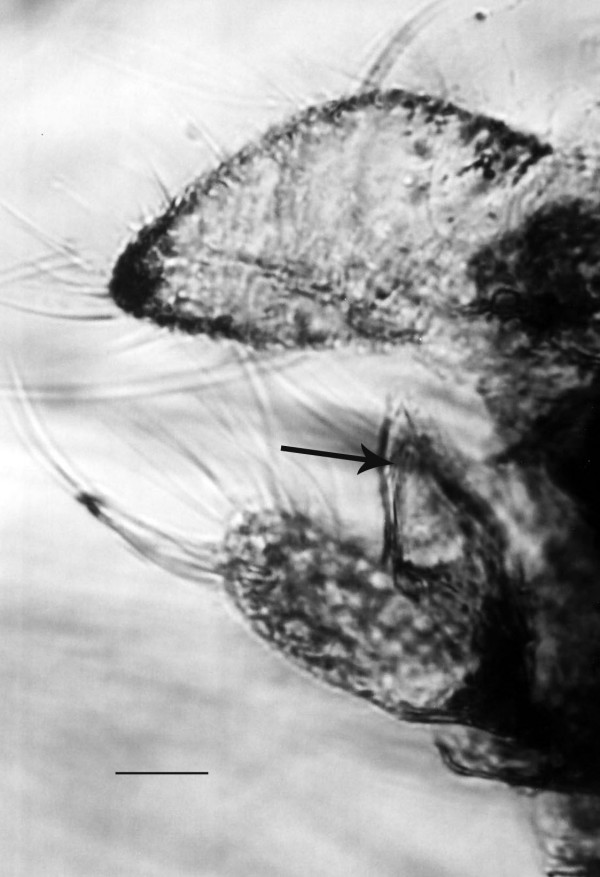
**Terminalia of *Lutzomyia adiketis*.** Arrow shows spatulate rods. Bar = 24 μm.

**Figure 4:**
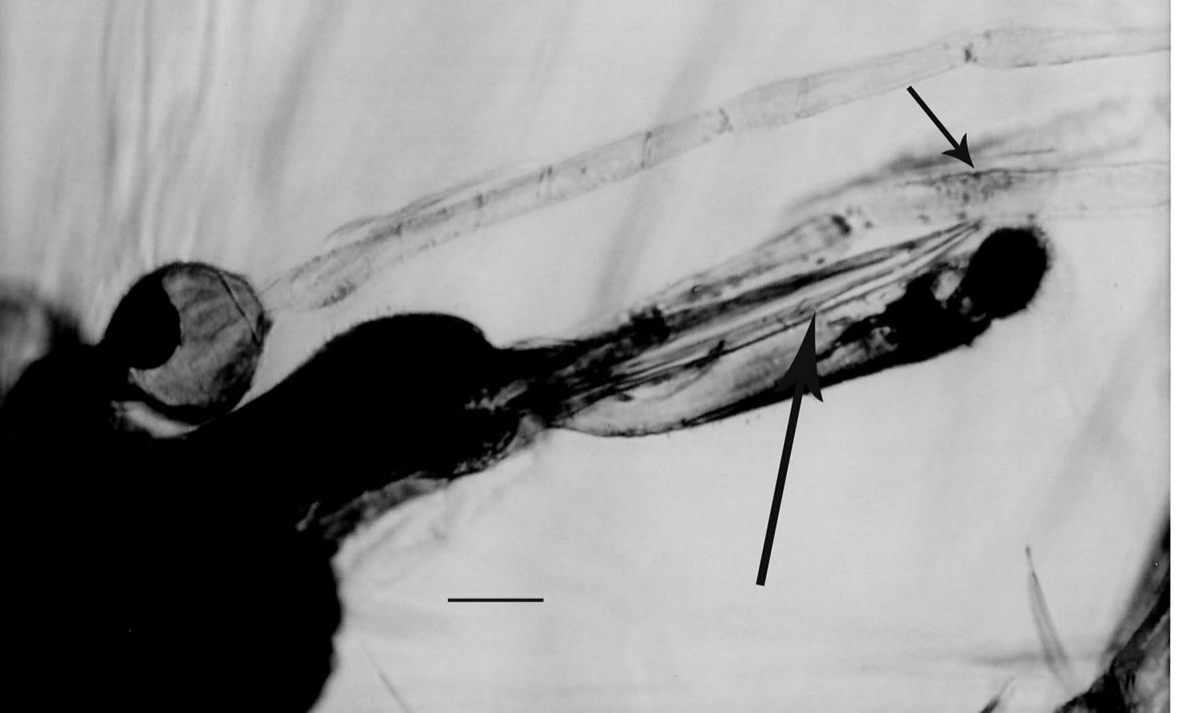
**Head of *Lutzomyia adiketis*.** Small arrow shows Newstead's scales on base of third palpomere. Large arrow shows mandibles. Bar = 32 μm.

***Lutzomyia adiketis ***sp.n. (Figs. 1, 2, 3, 4)

Length = 1.3 mm; body, legs and antennae light brown.

Head; Length, 315 μm; eye bridge absent; length of proboscis, 202 μm; maxillary palp extending well beyond tip of proboscis; length of maxillary palp, 544 μm; palpal formula 1-4-2-3-5; Newstead's scales in oval area on basal half of 3^rd ^palpomere; lengths of palpomeres; 1, 44 μm; 2, 89 μm; 3, 120 μm; 4, 82 μm; 5, 209 μm; ratio of palp segments, 1/2 = 0.49; 2/5 = 0.43; 4/2 = 0.64; 1/5 = 0.21; 3/5 = 0.57; 4/5 = 0.39; 1/3 = 0.37; flagellomeres 14, fusiform; scape short, stout; pedicel globular, terminal 3 segments subequal, shorter and broader than remainder; lengths of antennomeres (in microns) 1, 38; 2, 63 (60); 3, 171 (171); 4, 92 (92); 5, 82 (89); 6, 86 (79); 7, 82 (82); 8, 79 (76); 9, 86 (82); 10, 82 (82); 11, 76 (79); 12, 75 (83); 13, 81 (68); 14, 51 (57); 15, 41 (48); 16, 41 (41) (terminal spine on segment 16, 10 (10); ascoids absent on flagellomeres; cibarium not visible.

#### Thorax

Length = 441 μm ; wing with Sc forked, one branch reaching costa, other branch reaching R_1_; Rs four-branched with all branches extending to wing margin; Rs shorter than R _2-4_; R_1 _as long as R _2+3 _; R _2-4 _longer than R _2+3_; vein R_2 _longer than R_2+3 _; veins Sc, R_1_, R_2 _and R_3 _noticeably curved anteriorly at costa (R_1 _perpendicular to costa), R_5 _straight, veins M_1 _and M_2 _curved posteriorly at costa, vein CuA_2 _meets wing margin distal to R _2+3 _fork; termination of R_1 _distal to fork of R _2+3_; wing long and narrow, length 1.23 mm, width 0.30 mm, L/W = 4.1; wing values, α = 246, β = 211, δ = 18, γ = 243; wing ratio values, β/α, 0.86; γ/α, 0.98; δ/β, 0.09; β/γ, 0.87; membrane hyaline, main veins and wing surface bearing microtrichia; hind femora lacking teeth, length metafemur = 662, forefemur = 580; metatibia = 857, protibia = 580; length metatarsomeres, 1 = 454, 2 = 233, 3 = 158, 4 = 126, 5 = 76; length protarsomeres, 1 = 315 (328), 2 = 189 (183), 3 = 126 (126), 4 = 113 (107), 5 = 50 (50); tarsal claws paired, simple, thin, small, curved sharply at base, length 18–23 μm; spermatheca and associated reproductive structures not visible.

#### Abdomen

Abdomen extended, ten segments clearly visible; length, 800 μm; cerci 116 μm long and 54 μm wide; lobes on eight sternite 48 μm in length; spatulate rods 41 μm in length.

Male. – unknown

Type specimen. – Holotype female in Dominican amber deposited in the Poinar amber collection (accession # P-3–5) maintained at Oregon State University.

Etymology. – *adiketis *is from the Greek "adikos, which means injurious.

### Description of Trypanosomatid

Inside the alimentary tract of *L. adiketis *were hundreds of promastigotes of a trypanosomatid parasite, some of which had entered the hemocoel of the fly. Amastigotes, promastigotes and paramastigotes occurred in the proboscis of the sand fly. This trypanosomatid is provisionally described below as a new species in the genus *Paleoleishmania *Poinar & Poinar [6], a collective genus established for fossil digenetic trypanosomatids associated with sand flies.

Phylum Euglenozoa Cavalier-Smith 1981;

Class Kinetoplastea Honigberg 1963;

Order Kinetoplastida Honigberg 1963;

Family Trypanosomatidae Dolfein 1901

Genus *Paleoleishmania *Poinar & Poinar 2004

**Figure 5:**
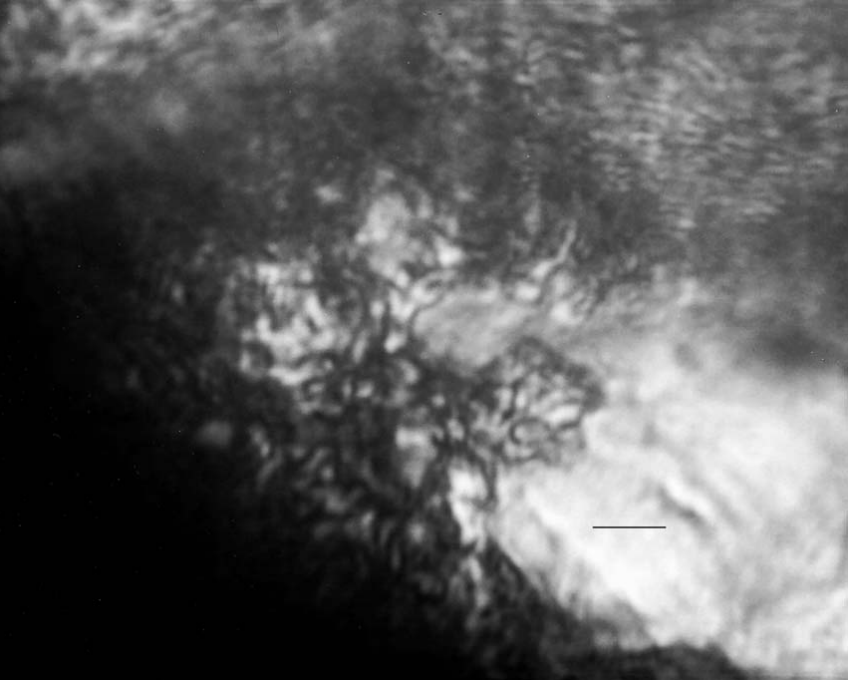
**Promastigotes of *Paleoleishmania neotropicum *in the foregut of *Lutzomyia adiketis*. **Bar = 7 μm.

**Figure 6:**
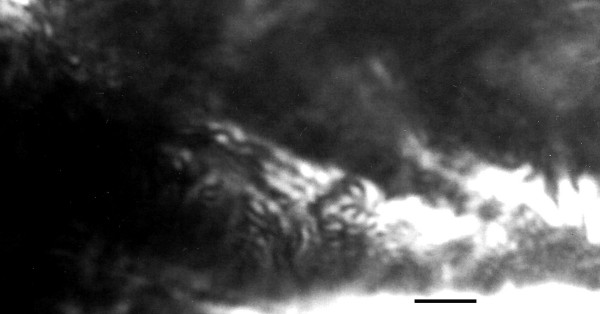
**Promastigotes of *Paleoleishmania neotropicum *in the midgut of *Lutzomyia adiketis*. **Bar = 7 μm.

**Figure 7:**
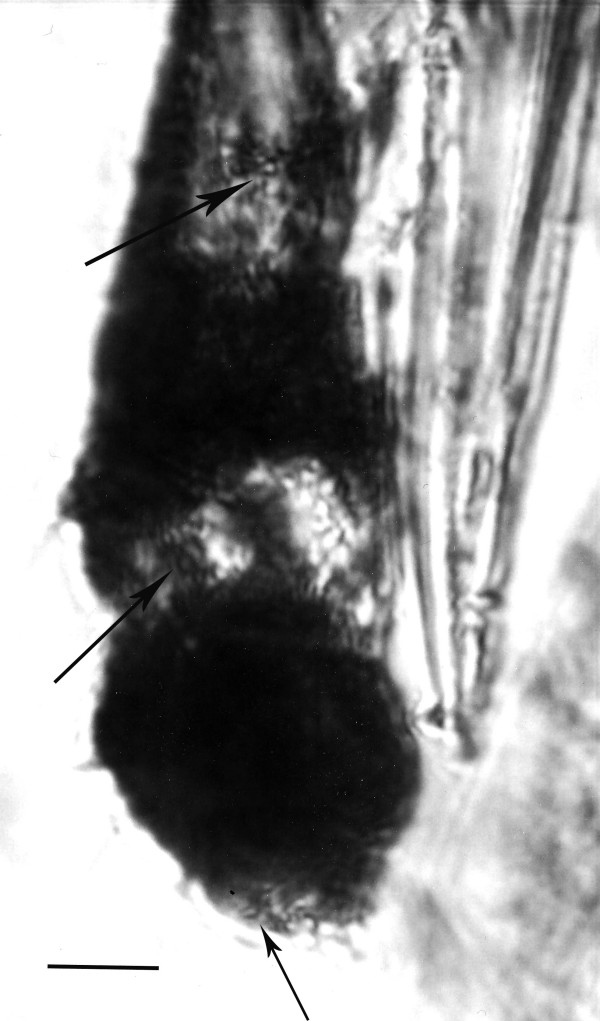
**Promastigotes of *Paleoleishmania neotropicum *in the proboscis (arrows) of *Lutzomyia adiketis*.** Bar = 20 μm.

**Figure 8:**
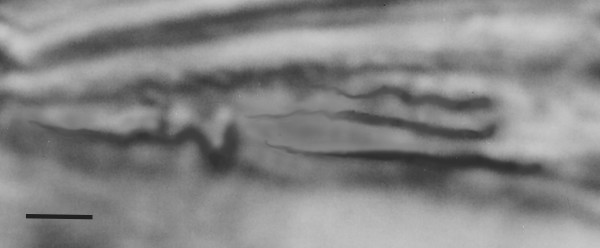
**Four promastigotes of *Paleoleishmania neotropicum *in the proboscis of *Lutzomyia adiketis*.** Bar = 4 μm.

**Figure 9:**
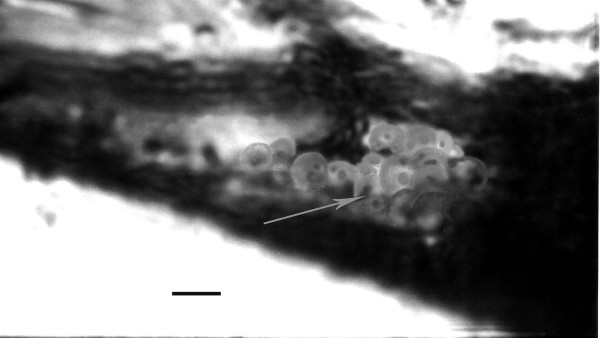
**Cluster of amastigotes (arrow) of *Paleoleishmania neotropicum *in the proboscis of *Lutzomyia adiketis*.** The large dark areas correspond to nuclei, while the smaller ones to kinetoplasts. Bar = 7 μm.

**Figure 10:**
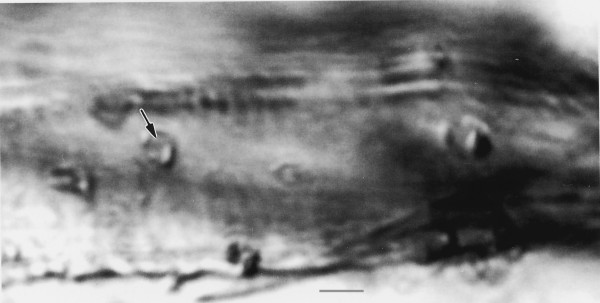
Amastigotes of *Paleoleishmania neotropicum *in the proboscis of *Lutzomyia adiketis*. Arrow shows amastigote with nucleus and kinetoplast. Bar = 7 μm.

**Figure 11:**
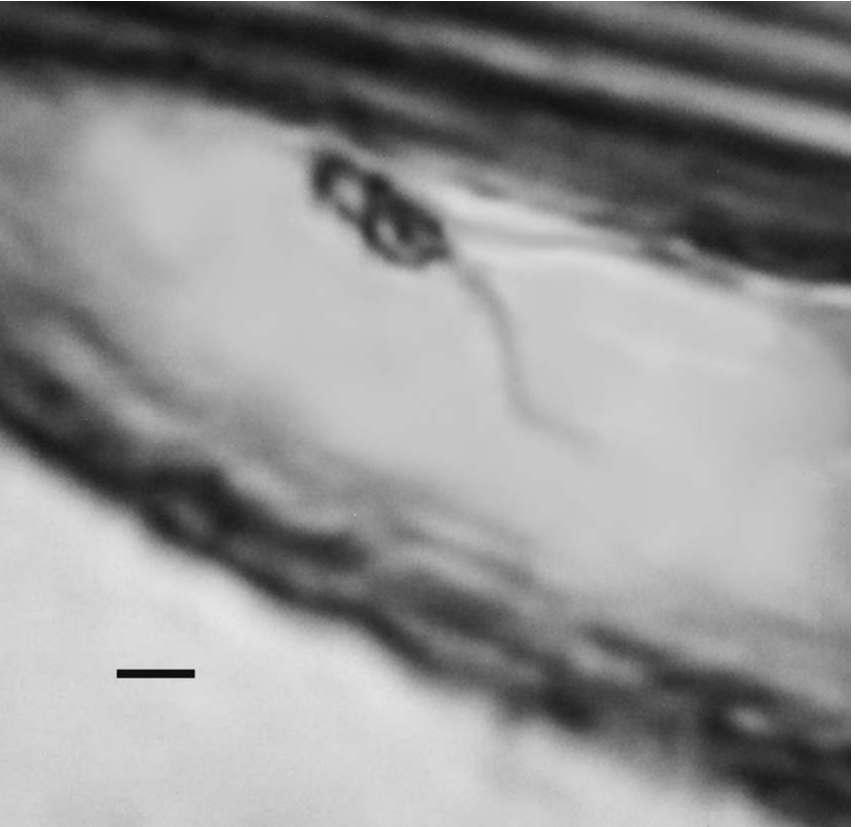
**Two paramastigotes of *Paleoleishmania neotropicum *in the proboscis of *Lutzomyia adiketis*.** Note long flagella. Bar = 5 μm.

*Paleoleishmania neotropicum *sp. n. (Figs. 5, 6, 7, 8, 9, 10, 11)

Promastigotes (N = 20) Flagellated stages containing nuclei and kinetoplasts; ranging from 6–10 μm in length, 1–3 μm in width and with flagella ranging from 6–11 μm in length (Figs. 5, 6, 7, 8).

Amastigotes (N = 20) Spherical to oval, containing nuclei and kinetoplasts, ranging between 4–7 μm in greatest diameter (Figs. 9, 10).

Paramastigotes (N = 4) Oval, relatively short, 3–5 μm in length and 2–4 μm in width, with adjacent nuclei and kinetoplasts; flagella long (10–20 μm)(Fig. 11).

#### Locality

Amber mine in the Cordillera Septentrional of the Dominican Republic.

#### Host

The insect host, *Lutzomyia adiketis *(Diptera: Phlebotomidae), is described in the present work. The vertebrate host is unknown.

## Discussion

### Sand fly vector

The absence of an eye bridge, fusiform flagellomeres, Rs four branched, 2 longitudinal veins present between the radial and medial forks and A_1 _absent, place the fossil in the Phlebotomidae [2,8]. The species shares many characters found in the extant subgenera *Lutzomyia *França and *Pintomyia *Costa Lima 1932, however the latter genus is characterized by a row of spines on the femur, which the fossil does not possess, thus it is tentatively assigned to the subgenus *Lutzomyia*. *Lutzomyia adiketis *differs from all previously described Dominican amber sand flies by its forked Sc vein, a previously used diagnostic character [3-5] and a character that occurs on some members of the subgenus *Lutzomyia *[9].

Four different configurations of the Sc vein exist in sand flies [9]. The Sc vein can be free, with the distal end not connected to either the costa or R1, as occurs in the Dominican amber *Pintomyia paleotownsendi *Andrade Filho et al. [10] and *Pintomyia falcaorum *Brazil et al. [11], Sc can meet the costa vein, as in the Dominican amber *P. paleotrichia *Andrade Filho et al. [12] or the Sc can meet R_1_, as in the Dominican amber *Pintomyia brazilorum *Andrade Filho et al. [13], *P. killickorum *Andrade Filho et al. [14] and all five species described by Peñalver & Grimaldi [15]. The fourth condition, where Sc forks distally, uniquely occurs only on *L. adiketis*, among the described Dominican amber fossils.

In addition to the forked Sc vein, the lengths of the papal segments, especially the second, fourth and fifth, the ratio of palpal segment 1 to palpal segment 2, the length/width ratio (4.1) of the wing and values of α, β, δ, γ, on *L. adiketis *differ from corresponding values of the five species of Peñalver & Grimaldi [15]. In order to exclude intraspecific variation due to size alone, ratios were found to be more useful than the actual values in several cases. Thus the ratio β/α (0.86) in *L. adiketis *(compared to a range of 0.57–0.71 in the species of Peñalver & Grimaldi [15]) and the ratio δ/β, (0.09) in *L. adiketis *(compared to the range of 0.14–0.38 in the species of Peñalver & Grimaldi [15]) distinguishes *L. adiketis *from the latter species.

Some extant sand flies in the subgenus *Lutzomyia *also have a forked Sc vein [9], however they can be distinguished from *L. adiketis *by the following characters. In *Lutzomyia alencari *Martins, Souza & Falcão, vein R_1 _meets the costa at the same distance from the wing base as CuA_2 _meets the wing margin. In *L. adiketis*, CuA_2 _meets the wing margin distal to the termination or R_1_. The wing ratio δ/β can be used to separate *L. adiketis *(0.09) from *L. gasparviannai *Martens, Godoy & Silva (0.57), *L. ischyracantha *Martens, Falcão & Silva (0.00) and *L. ischnacantha *Martins, Sousa & Falcão (0.89). The ratio of the length of R _2+3 _to R _2 _in *L. gaminarai *(Cordero, Vogelsong & Cossio)(0.86) is larger than the same ratio in *L. adikites *(0.74).

There are only two extant species of *Lutzomyia *found in the Dominican Republic [*L. cayennensis hispaniolae *(Fairchild & Trapido) and *L. christophei *(Fairchild & Trapido)], both of which belong to the *Verrucarum *species group [9]. None of the species in this group have forked Sc veins, which separates them from *L. adiketis*.

It is unfortunate that characters of the female external genitalia are so little used in the systematics of the group, even though their diagnostic importance was demonstrated by Mukhopadhyay and Ghosh [16]. The size and shape of the cerci, lobes on the 8^th ^sternite and spatulate rods on the ninth sternite could serve as diagnostic characters. The spatulate rods on *L. adiketis *are quite distinctive and similar rods have not been observed on any other amber sand flies examined by the author.

Extant species of *Lutzomia *are restricted to the New World and their host range is quite extensive, including over 30 families of mammals, birds, reptiles and amphibians [17]. Several extant members of the subgenus *Lutzomyia *feed on humans and are proven vectors of *Leishmania infantum chagasi*, the causal agent of American visceral leishmaniasis [9]. The vertebrate host of *L. adiketis *is unknown.

### Trypanosomatid

A single, anteriorly directed flagellum, compact kinetoplast and nucleus places *P. neotropicum *in the family Trypanosomatidae. The presence of amastigotes is evidence that *L. neotropicum *is digenetic, since in *Leishmania*, amastigotes are only formed in the vertebrate host and there are no known monogenetic flagellates of sand flies [18]. Paramastigotes (metacyclic stages), which are produced inside the gut of the sand fly, also are only infective to vertebrates [18-23]. The flagella always emerged from the anterior end of the flagellates and were not attached to the body along part of most of their length by undulating membranes. Thus, it is unlikely that the fossils belong to the monogenetic genus *Blastocrithidia *Laird, since the epimastigote stage dominates the life cycle and it has never been recovered from sand flies [18]. Species of *Endotrypanum *Mensal & Brimont, which are vectored by sand flies and form promastigotes and amastigotes in the vector [18], are restricted to sloths today and all sloth remains in Hispaniola are from Quaternary cave deposits [24]. If sloths were absent in Hispaniola during the mid-Tertiary when Dominican amber was formed, it would have been impossible for *Lutzomyia adiketis *to acquire *Endotrypanum*. It is also unlikely that the flagellates belong to the related genus *Phytomonas *Donovan since extant species only occur naturally in plant-feeding bugs (Hemiptera)[18].

No vertebrate blood cells were found in the sand fly, however these would be very difficult to detect in the thoracic gut of the intact insect. The round amastigotes of *Paleoleishmania neotropicum *are similar in size (4 – 7 μm) and morphology to those of extant species of *Leishmania *[19-21,23]. Since amastigotes are normally ingested with the blood meal and pass into the midgut of feeding sand flies, the amastigotes in the fossil proboscis may have arrived in that location immediately after the fossil sand fly finished feeding and became entombed in resin. The amastigotes may even have been multiplying in that location since in *Leishmania chagasi*, a cycle of amastigote division may occur before or even concurrently with the transformation of amastigotes into promastigotes [21]. Another possibility is that the amastigotes were regurgitated into the proboscis while the sand fly was struggling to escape from the resin.

Promastigotes of extant *Leishmania *spp. vary from 6 to 24 μm in length (excluding the flagellum) [19-21], which are within the size range of those reported here (6–10 μm). The promastigotes in the proboscis of *L. adiketis *(Fig. 9) could have developed from amastigotes or they could be "infective promastigotes", similar to those of *Leishmania chagasi*, which occur in the mouthparts (ventral surface of the labrum-epipharynx) of *Lutzomyia longipalpis *[21]. However, it is also possible that the promastigotes in the proboscis were acquired directly from the vertebrate, since in reptilian hosts, the stages imbibed can be either amastigotes and/or promastigotes [25,26].

The paramastigotes in the proboscis of *L. adiketis *(Fig. 11) probably developed from an earlier blood meal. Female sand flies are capable of living a month or more [17] and normally engorge blood at least twice during their lifetime, with a batch of eggs laid after each blood meal [25].

The main diagnostic characters of *P. neotropicum *at this time are the structure of the amastigotes, promastigotes and paramastigotes, and its association with the extinct sand fly, *Lutzomyia adiketis*, in Dominican amber.

Fossil evidence has provided a possible scenario of how sand fly-trypanosomatid associations evolved [28-30]. Free-living trypanosomatids that were associated with a fungal food source also occurred in the alimentary tract of a sand fly larva in Burmese amber. It is postulated that these flagellates were carried transtadially into the adult stage and then transmitted to vertebrates. The establishment of the parasites in the vertebrate and their subsequent re-acquisition by adult sand flies is undoubtedly a rare event and would only occur under ideal conditions. It is unknown whether *Leishmania *originated in the New or Old World [31,32]. If the above evolutionary pattern of flagellate acquisition is correct, different strains of trypanosomatids could have appeared at different localities and times over the past 100 or so million years. The 100 million year-old Burmese amber sand fly-trypanosomatid, *P. proterus *[6,7] undoubtedly arose independently from *P. neotropicum*, which could well be the progenitor to one or more of the Neotropical *Leishmania *clades.

## Conclusion

Fossil vertebrate parasites inside insect vectors in amber provide us with a time and place record of diseases affecting terrestrial organisms [27,29,33,34].

The present study provides the first fossil evidence that Neotropical sand flies were vectors of trypanosomatids in the mid-Tertiary. *Lutzomyia adiketis*, in Dominican amber, is an extinct species of sand fly that was carrying the trypanosomatid *Paleoleishmania neotropicum*. It is possible that *P. neotropicum *is a progenitor of at least one of the several extant Neotropical *Leishmania *clades.

## Methods

### Specimens

The amber piece containing the fossil sand fly with the trypanosomatids is oval in outline, measuring 18 mm along the long axis, 12 mm along the short axis and 2 mm in thickness. The sand fly is well preserved but both middle legs and the left hind leg are detached, as are almost all hairs from the antennae. Remains of these, along with many body hairs, are positioned behind the fossil, indicating that the sand fly struggled forward to free itself from the resin. The alimentary track was ruptured, allowing some flagellates in the gut to leak into the hemocoel. Observations, drawings and photographs were made with a Nikon SMZ-10 R stereoscopic microscope and Nikon Optiphot compound microscope (with magnifications up to 1050×). Some of the photographs were enhanced in Adobe Photoshop.

### Locality

The amber containing the fossils was mined in the northern mountain range (Cordillera Septentrional) of the Dominican Republic between Puerto Plata and Santiago. Dating of Dominican amber is controversial, with the youngest proposed age of 20–15 mya based on foraminifera [35] and the oldest as 45–30 mya based on coccoliths [36]. Most of the amber is secondarily deposited in turbiditic sandstones of the Upper Eocene to Lower Miocene Mamey Group [37].

### Source

Dominican amber was formed by the extinct legume tree, *Hymenaea protera *Poinar [38] and the original landscape was considered to be a moist, tropical forest [39]. Terminology in the description follows that presented in the "Manual of Nearctic Diptera" [40].

## Declaration of competing interests

The author declares that he has no competing interests.
